# ICAM-2 Expression Mediates a Membrane-Actin Link, Confers a Nonmetastatic Phenotype and Reflects Favorable Tumor Stage or Histology in Neuroblastoma

**DOI:** 10.1371/journal.pone.0003629

**Published:** 2008-11-03

**Authors:** Karina Jin Yoon, Doris A. Phelps, Rebecca A. Bush, Joanna S. Remack, Catherine A. Billups, Joseph D. Khoury

**Affiliations:** 1 Department of Molecular Pharmacology, St. Jude Children's Research Hospital, Memphis, Tennessee, United States of America; 2 Department of Biostatistics, St. Jude Children's Research Hospital, Memphis, Tennessee, United States of America; 3 Department of Pathology, St. Jude Children's Research Hospital, Memphis, Tennessee, United States of America; Dresden University of Technology, Germany

## Abstract

The actin cytoskeleton is a primary determinant of tumor cell motility and metastatic potential. Motility and metastasis are thought to be regulated, in large part, by the interaction of membrane proteins with cytoplasmic linker proteins and of these linker proteins, in turn, with actin. However, complete membrane-to-actin linkages have been difficult to identify. We used co-immunoprecipitation and competitive peptide assays to show that intercellular adhesion molecule-2 (ICAM-2)/α-actinin/actin may comprise such a linkage in neuroblastoma cells. ICAM-2 expression limited the motility of these cells and redistributed actin fibers *in vitro*, and suppressed development of disseminated tumors in an *in vivo* model of metastatic neuroblastoma. Consistent with these observations, immunohistochemical analysis demonstrated ICAM-2 expression in primary neuroblastoma tumors exhibiting features that are associated with limited metastatic disease and more favorable clinical outcome. In neuroblastoma cell lines, ICAM-2 expression did not affect AKT activation, tumorigenic potential or chemosensitivity, as has been reported for some types of transfected cells. The observed ICAM-2-mediated suppression of metastatic phenotype is a novel function for this protein, and the interaction of ICAM-2/α-actinin/actin represents the first complete membrane-linker protein-actin linkage to impact tumor cell motility *in vitro* and metastatic potential in an *in vivo* model. Current work focuses on identifying specific protein domains critical to the regulation of neuroblastoma cell motility and metastasis and on determining if these domains represent exploitable therapeutic targets.

## Introduction

The actin network is a primary determinant of the motility of cells and organelles. This complex network regulates the movement of amoebae, distribution of bacteria within cells, cell migration in embryogenesis and angiogenesis, and the migratory and invasive (metastatic) potential of tumor cells. In tumor cells, motility and metastasis are thought to be regulated at least in part by the interaction of membrane-bound proteins with the actin cytoskeletal network [1]–[5]. Multiple signaling pathways and protein interactions modulate the motility and migration of tumor cells [3]–[8], but specific molecular interactions that control the complicated process of metastasis have been difficult to identify. In normal cells, the interaction of a particular membrane-bound cell adhesion molecule with a specific cytoplasmic linker protein has been associated with a unique cell function. In inactive neutrophils, for example, membrane-bound β_2_-integrin binds to the cytoplasmic linker protein talin. In contrast, in activated neutrophils β_2_-integrin binds to an alternate linker protein, α-actinin [9]. While both talin and α-actinin can bind directly to actin, it is not yet known how the association of β_2_-integrin with each linker proteins affects their binding to actin.

In tumor cells, members of the ezrin-radixin-moesin (ERM) family of cytoplasmic actin cytoskeletal linker proteins play critical roles in the regulation of metastatic potential. ERM linker proteins, as well as α-actinin, merlin, and band 4.1 linker proteins [4]–[6], [10]–[12], have been implicated in tumor progression or in suppression of the metastatic potential of tumor cells. Cell adhesion molecules (CAMs) to which these linker proteins bind, such as deleted in colon cancer (DCC) and carcinoembryonic antigen-related cell adhesion molecule 1 (CEACAM1), are members of the IgG superfamily to which ICAM-2 belongs. DCC and CEACAM1 contribute to the adhesive, migratory, or invasive properties of several types of solid tumor cells [13]–[16] including neuroblastoma [17], but the molecular interactions that mediate these functions have been difficult to identify. The data herein suggest that in neuroblastoma cells the association of ICAM-2 with α-actinin and actin may represent such an interaction.

Other functions have also been reported for ICAM-2. Particularly well characterized are its roles as a co-stimulatory signal for T cell stimulation [18] and in mediating neutrophil adhesion and migration through endothelial cell monolayers [19]–[21]. These roles have been documented by *in vivo* [22]–[24] as well as *in vitro* methods. In specific cell types ICAM-2 also activates the PI3Kinase/AKT pathway [25], [26] and promotes cell migration [27], suggesting that this adhesion molecule has multiple, apparently unrelated functions. Neither the expression nor the function of ICAM-2 in neuroblastoma cells has been investigated previously.

Neuroblastoma is a pediatric tumor thought to arise from cells of the neural crest. Patients diagnosed with neuroblastoma frequently achieve complete remissions, but up to 80% of high-risk patients develop metastatic disease that does not respond to therapy [28]. As is true for most types of solid tumors, the primary cause of death is the development of metastatic disease; but no current therapies specifically target the metastatic process, as a potential approach for limiting the occurrence or the progression of disseminated tumors. The integral role of cell motility (migration and invasion) in the metastatic process and data in the literature suggesting the potential involvement of CAMs in regulating cell motility provided the rationale for the studies reported here. The long-range goal of the work is to identify molecular interactions that regulate the metastatic potential of neuroblastoma, and to determine if these molecular interactions represent potential therapeutic targets.

## Results

### ICAM-2 associates with α-actinin and actin to establish a membrane-to-actin linkage

In tumor cells, proteins of the cytoplasmic actin linker families contribute to tumorigenesis and metastasis. RT-PCR analysis of a panel of neuroblastoma cell lines (data not shown) showed that neuroblastoma cells express two of these linker proteins: ezrin and α-actinin. Ezrin and α-actinin bind to the intracellular domain of the intercellular adhesion molecule-2 (ICAM-2) [29], [30] and to actin. Therefore, we postulated that membrane-bound ICAM-2 binds to cytoplasmic ezrin or α-actinin which, in turn, binds to actin to regulate the motility—and hence the metastatic potential—of neuroblastoma cells.

**Figure 1:**
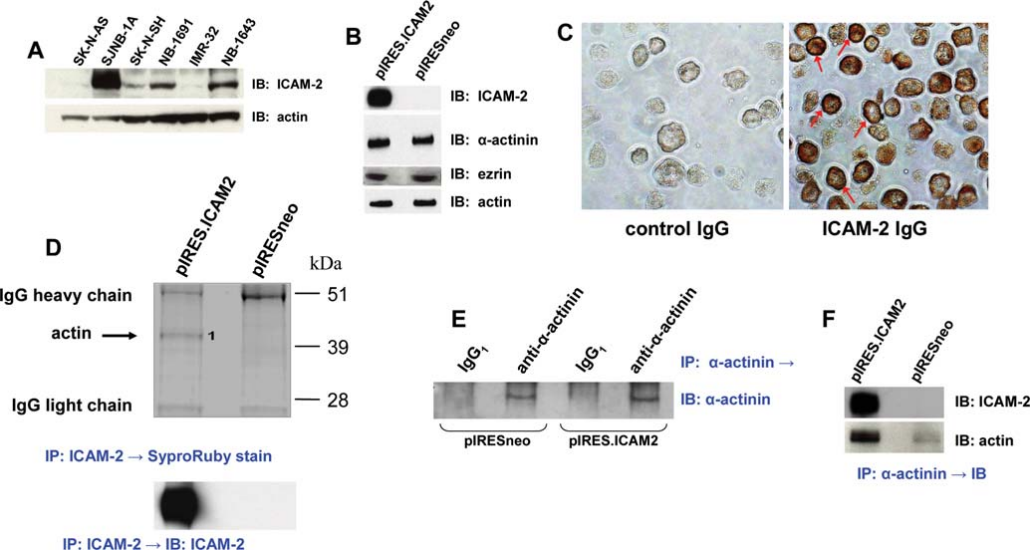
In neuroblastoma cells, membrane-bound ICAM-2 associates with α-actinin and actin. A) Immunoblot of whole cell lysates (35 µg protein/lane) of six neuroblastoma cell lines, comparing endogenous levels of expression of ICAM-2. B) Immunoblots of SK-N-AS cells transfected with control plasmid (pIRESneo) or with plasmid encoding ICAM-2 (pIRES.ICAM2) show that transfectants expressed similar levels of α-actinin, ezrin, and actin, but SK-N-ASpIRES.ICAM2 cells expressed a higher level of ICAM-2 than SK-N-ASpIRESneo cells (25 µg protein/lane). C) ICAM-2 expressed in SK-N-ASpIRES.ICAM2 cells localized to cell membranes (examples indicated by red arrows). D) Co-immunoprecipitation co-precipitation/mass spectroscopic analysis (actin) showed that actin co-precipitated with ICAM-2 in ICAM-2 transfectants. Band #1 was >99% identical to human actin. E) Immunoprecipitation/immunoblot showing equal amounts of α-actinin precipitated from lysates of SK-N-ASpIRESneo and SK-N-ASpIRES.ICAM2 cells. F) Immunoprecipitation/immunoblots showing that ICAM-2 and actin co-precipitated with α-actinin in lysates from SK-N-ASpIRES.ICAM2 cells, but little if any ICAM-2 and actin co-precipitated with α-actinin in SK-N-ASpIRESneo cells.

We first demonstrated using immunoblotting, that 6 of 6 neuroblastoma cells express ICAM-2 (Fig. 1A). ICAM-2 protein had the expected apparent mass of 55–60 kDa [31] in all six neuroblastoma cell lines (as well as other types of solid tumor cell lines [data not shown]), and the level of ICAM-2 expression varied with SJNB-1A>NB-1691, SK-N-SH and NB-1643>SK-N-AS and IMR-32.

We then transfected a cell line with low endogenous ICAM-2 expression (SK-N-AS) with control pIRESneo or with the ICAM-2-encoding plasmid, and compared the phenotypes of the stable congenic transfected cell lines. Immunoblots of whole cell lysates of SK-N-ASpIRESneo and SK-N-ASpIRES.ICAM2 cells confirmed that the transfectants expressed similar levels of actin, α-actinin, and ezrin, but differed in levels of ICAM-2 expression (Fig. 1B). As reported for normal endothelial cells [19], immunoperoxidase staining of paraffin-embedded cells showed that ICAM-2 in transfected SK-N-ASpIRES.ICAM2 cells localized to cell membranes (Fig. 1C).

Using these transfectants, we conducted co-immunoprecipitation assays to determine whether α-actinin or ezrin associated with ICAM-2 and/or actin in transfected neuroblastoma cells. Proteins in lysates from pIRESneo and pIRES.ICAM2 transfectants were immunoprecipitated with a monoclonal antibody to ICAM-2, and an aliquot of precipitated proteins was analyzed by immunoblot with a polyclonal antiserum to this protein. The blot in the lower panel of Fig. 1D shows the expected difference in level of ICAM-2 precipitated from ICAM-2 compared to control transfectants. A second aliquot of precipitated proteins was separated by electrophoresis and stained with SyproRuby to detect co-precipitated proteins. SyproRuby-stained gels showed a distinct band of ∼42 kDa, unique to the SK-N-ASpIRES.ICAM2 cells. This band (#1, in upper panel of Fig. 1D) was confirmed to be >99% identical to human actin by mass spectrometry, indicating that in SK-N-ASpIRES.ICAM2 cells, actin associated with ICAM-2. Co-immunoprecipitations were then done with antibodies to α-actinin, actin, or ezrin, and precipitated products identified by immunoblot. The immunoprecipitation/immunoblot performed with a monoclonal antibody to α-actinin or control immunoglobulin/polyclonal antiserum (Fig. 1E) showed that control precipitations were appropriately negative and that equal amounts of α-actinin were precipitated from SK-N-ASpIRESneo and SK-N-ASpIRES.ICAM2 transfectants. Subsequent immunoblots to detect proteins that co-precipitated with α-actinin revealed that both actin and ICAM-2 associated with α-actinin in lysates from SK-N-ASpIRES.ICAM2 cells, but little or no actin or ICAM-2 co-precipitated with α-actinin in lysates from SK-N-ASpIRESneo cells (Fig. 1F). Data in Fig. 1F also suggested that little actin associated with α-actinin in the absence of ICAM-2 expression. The specificity of the observed association of ICAM-2, α-actinin, and actin was verified by immunoprecipitating ICAM-2 and immunoblotting for ICAM-2, α-actinin, or actin (Supporting Information, Fig. S1). Ezrin was not detected to co-precipitate with ICAM-2 or with actin in either cell line, nor was actin or ICAM-2 detected to co-precipitate with ezrin (data not shown).

These co-immunoprecipitation data showed that ICAM-2 associated with α-actinin and actin in neuroblastoma cell lysates. We note that the data are qualitative and do not necessarily reflect the ratio in which these proteins associate. ICAM-2 is a heavily glycosylated protein that exists in multiple forms that migrate as a diffuse band having an area×density signal approximately 300%–400% greater than that for the same amount of de-glycosylated ICAM-2. Further, α-actinin induces actin self-association [32], resulting in variable numbers of actin molecules associating with a single α-actinin molecule. Nevertheless, the data indicate that ICAM-2, α-actinin, and actin associate with each other in neuroblastoma cell lysates.

### ICAM-2-mediated redistribution of actin fibers is disrupted by competitive peptides to inhibit ICAM-2/α-actinin or α-actinin/actin binding, and is associated with decreased tumor cell motility

The observed ICAM-2/α-actinin/actin association in cell lysates suggested that ICAM-2 expression in neuroblastoma cells might affect the function of the actin cytoskeleton. Alternatively, in other types of transfected cells, ICAM-2 has been reported to stimulate immune responses [22] and affect AKT phosphorylation and chemosensitivity [25]. Therefore, we evaluated the effect of ICAM-2 expression by SK-N-AS cells on AKT phosphorylation and sensitivity to vincristine, doxorubicin, or etoposide, and on actin fiber distribution and cell motility, using *in vitro* assays in which immune stimulation was not a potential factor.

#### Effect of ICAM-2 expression on AKT phosphorylation, drug sensitivity, and growth characteristics

**Figure 2:**
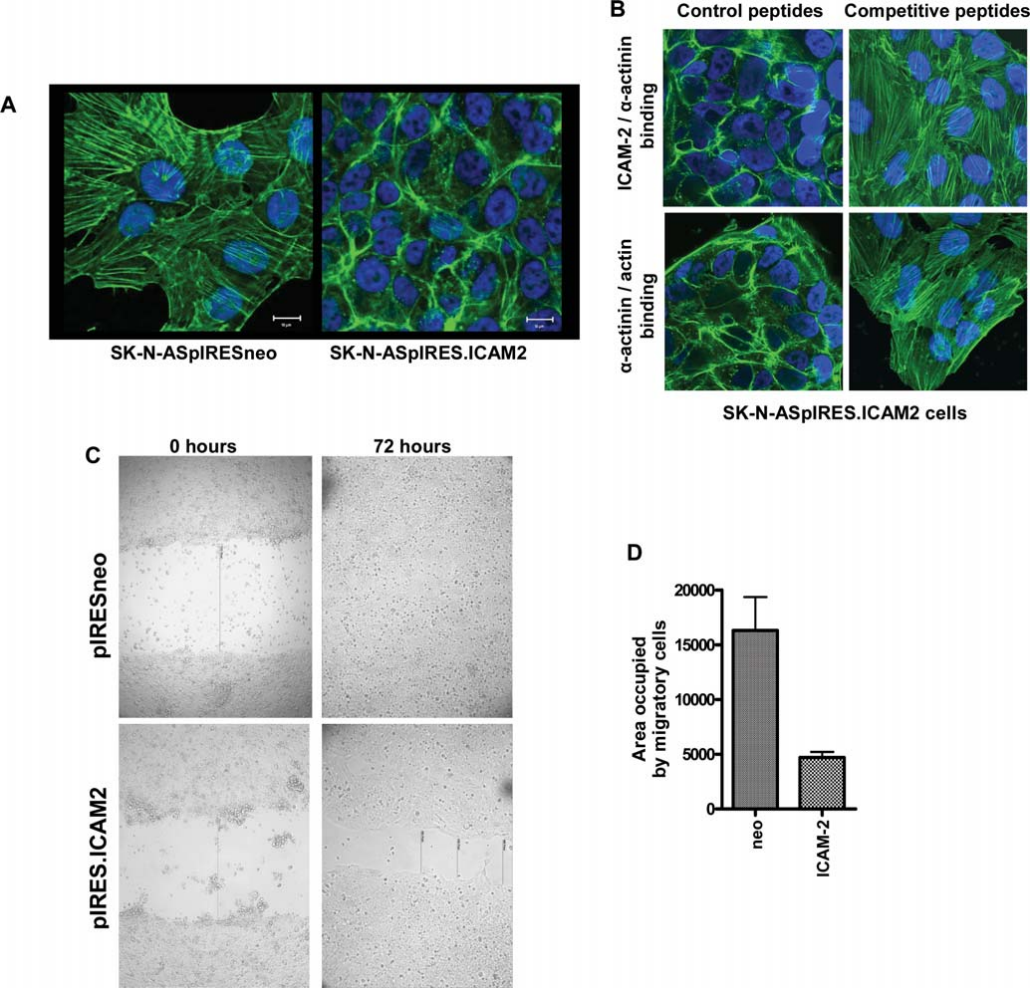
ICAM-2 expression affected the subcellular distribution of actin fibers and cell motility in two types of *in vitro* assays. A) Confocal fluorescence microscopic images of actin fibers visualized by FITC-phalloidan (green) staining demonstrate that ICAM-2 expression redistributed F-actin from a transverse to a juxtamembrane localization. Cell nuclei were visualized using DAPI (blue). B) Competitive peptides to inhibit ICAM-2/α-actinin binding or α-actinin/actin binding reversed the ICAM-2-mediated juxtamembrane distribution of actin fibers in neuroblastoma cells. C) In scratch assays, ICAM-2 transfectants migrated more slowly than control transfectants. The width of scratches at 0 hours for pIRESneo and pIRES.ICAM2 transfectants were 1127 and 1044 pixels, respectively. At 72 hours, no gap remained in wells containing SK-N-ASpIRESneo cells, while a scratch distance of ∼450 pixel width remained in wells containing SK-N-ASpIRES.ICAM2 cells. D) In modified Boyden chamber assays, ICAM-2 expression inhibited invasion of SK-N-AS cells through Matrigel to the distal side of porous membranes, as detected by Diff-Quick (IMEB, Inc., San Marcos, CA), and quantitated using an Alpha Imager software, as detailed in Materials and Methods.

In contrast to published reports, we did not detect ICAM-2-mediated increases in AKT phosphorylation as determined with a Ser473-specific antibody on immunoblots, or in sensitivity to etoposide or doxorubicin (data not shown). Neither did we detect ICAM-2 mediated-effects on cell cycle distribution, cell volume, or cell doubling time (data not shown). ICAM-2 expression did affect the morphology of cells as adherent monolayers. Adherent cells expressing ICAM-2 appeared larger and less spheroid in shape than cells expressing little or no ICAM-2 (evident in Fig. 2A).

#### Effect of ICAM-2 expression on actin fiber distribution and cell motility

FITC-phalloidan staining showed that, ICAM-2 redistributed intracellular actin fibers from a transverse to a juxtamembrane localization (Fig. 2A). This redistribution was observed irrespective of cell density (data not shown). We reasoned that if redistribution depended on the interaction of ICAM-2 with α-actinin and actin, peptides designed to competitively inhibit these interactions would be predicted to reverse the observed actin fiber redistribution pattern in SK-N-ASpIRES.ICAM2 transfectants. Consistent with this hypothesis, fibers reverted to a transverse distribution in SK-N-ASpIRES.ICAM2 cells exposed to each competitive peptide (Fig. 2B), but remained co-localized with cell membranes in cells exposed to scrambled control peptides. Similar results were also observed with NB-1691 neuroblastoma cells transfected with pIRESneo or pIRES.ICAM2 (data not shown).

Co-incident with observed ICAM-2-mediated redistribution of actin fibers was an inhibition of tumor cell motility (Figs. 2C and D), as assessed with two types of *in vitro* assays: scratch (wound) assays and modified Boyden chamber invasion assays. In scratch assays, cell monolayers were overlaid with Matrigel immediately after scratching, and the original scratch width was recorded in pixels. Photomicrographs taken at 0 hours and 72 hours (Fig. 2C) showed initial scratch widths of ∼1125 pixels and ∼1050 pixels for the pIRESneo and pIRES.ICAM2 transfectants, respectively. At 72 hours pIRESneo transfectants had completed closed the scratch, while the SK-N-ASpIRES.ICAM2 cells remained separated by a width of ∼450 pixels. In the second type of motility assay, a modified Boyden chamber invasion assay, significantly fewer SK-N-ASpIRES.ICAM2 cells migrated through a layer of Matrigel to the distal side of porous membranes (*P*<0.0003, Fig. 2D), compared to SK-N-ASpIRESneo cells. ICAM-2 expression also inhibited the motility of NB-1691 cells transfected with pIRESneo and pIRES.ICAM2 in this invasion assay (*P*<0.001, data not shown). Inhibition of tumor cell motility by ICAM-2 has not been reported previously. Most notably, *in vitro* results of phalloidan staining and cell motility assays demonstrated a function for ICAM-2 independent of its known immune stimulatory effect.

### ICAM-2-mediated redistribution of actin fibers and inhibition of tumor cell motility depended on an intact intracellular domain

**Figure 3:**
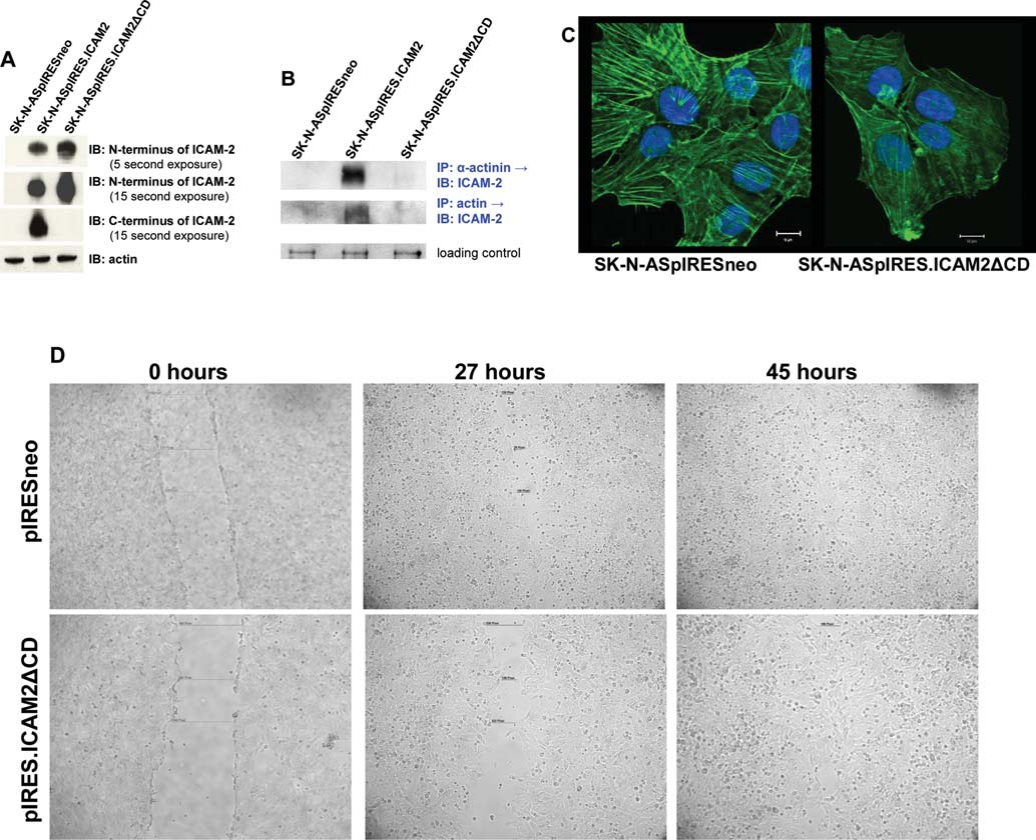
The effect of ICAM-2 on actin distribution and cell motility depends on an intact intracellular domain. A) SK-N-AS cells were transfected to express a truncated form of ICAM-2 with a deleted cytoplasmic domain (ICAM2ΔCD). Expression of full length ICAM-2 and ICAM2ΔCD was similar in respective transfected cell lines. B) ICAM-2, α-actinin, and actin co-precipitated only in transfectants expressing full length ICAM-2. C) ICAM2ΔCD had no effect on actin fiber distribution. (Compare with Fig. 2A). D) ICAM2ΔCD did not affect migration of SK-N-AS cells in scratch assays. Pixel width of scratches for pIRESneo and pIRES.ICAM2ΔCD transfectants at 0 hours and 27 hours were similar. No gap remained for either cell line 45 hours after scratch.

To then corroborate observations with competitive peptides indicating that ICAM-2/α-actinin binding affected actin fiber distribution, we transfected SK-N-AS cells to express a truncated form of ICAM-2. ICAM-2 and ICAM-2ΔCD (ICAM-2 deleted cytoplasmic domain) transfectants expressed similar levels of their respective forms of ICAM-2 (Fig. 3A) and both forms of this protein localized to cell membranes (Figs. 1C and S2), but ICAM2ΔCD lacked the 26-amino acid intracellular domain to which α-actinin binds. If ICAM-2/α-actinin binding were essential to the observed ICAM-2-mediated redistribution of actin fibers, expression of ICAM2ΔCD would have no effect on actin fiber distribution and, similar to SK-N-ASpIRESneo cells, actin fibers in SK-N-ASpIRES.ICAM2ΔCD cells would be in a transverse configuration. Immunoprecipitation/immunoblot experiments using the antibody that recognized the N-terminus of ICAM-2 (Fig. 3B) demonstrated that neither α-actinin nor actin associated with ICAM-2 in control transfectants or in transfectants expressing the truncated form of ICAM-2, and FITC-phalloidan staining (Fig. 3C) confirmed that expression of the truncated form of ICAM-2 lacking the α-actinin binding domain did not affect actin fiber distribution. Consistent with these observations, SK-N-pIRESneo and pIRES.ICAM2ΔCD transfectants migrated at similar rates in scratch assays, closing initial scratch widths of ∼525 pixels within 45 hours (Fig. 3D). The data show that ICAM-2-mediated redistribution of actin fibers and inhibition of tumor cell motility depended on an intact intracellular domain.

### ICAM-2 did not affect growth of neuroblastoma cells as subcutaneous xenografts, but did inhibit development of disseminated neuroblastoma tumors in a mouse model of metastatic neuroblastoma

**Figure 4:**
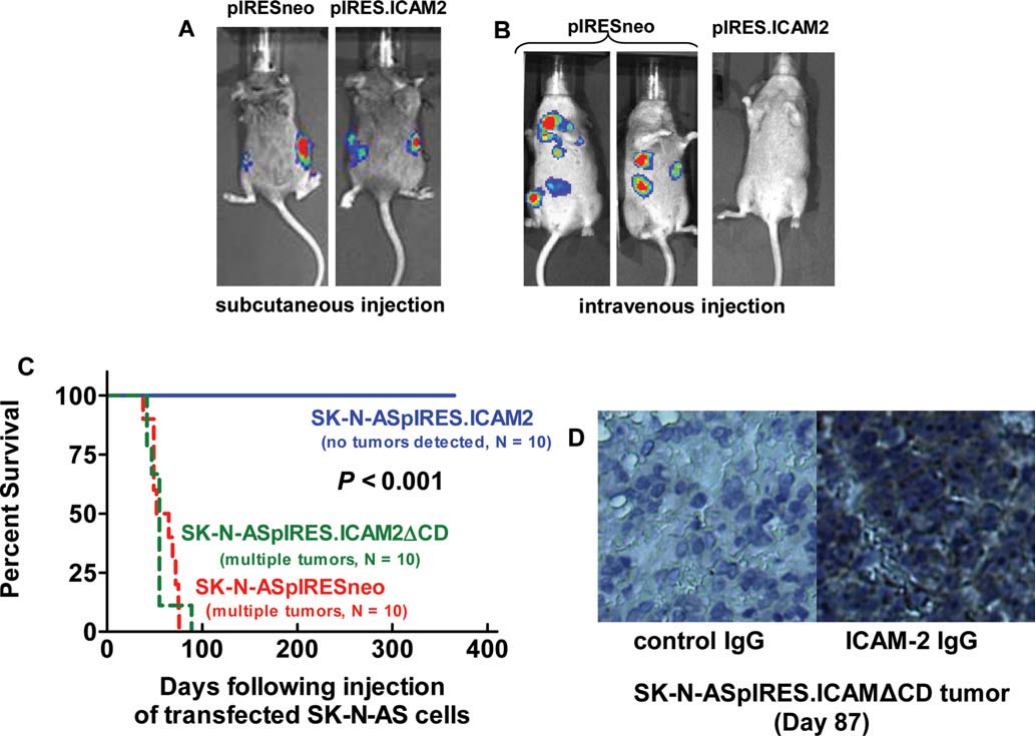
ICAM-2 expression inhibited the development of disseminated tumors but did not affect establishment or development of local subcutaneous tumors. A) ICAM-2 did not affect the growth of neuroblastoma cells as subcutaneous tumors in SCID mice, a measure of tumorigenic potential. B) ICAM-2 prevented the development of disseminated tumors, following intravenous tail vein injection of neuroblastoma cells, a measure of metastatic potential. C) Mice injected intravenously with SK-N-ASpIRES.ICAM-2 cells survived significantly longer (*P*<0.001) than mice receiving pIRESneo or pIRES.ICAM2ΔCD transfected cells. D) The failure of ICAM2ΔCD expression to suppress development of disseminated tumors was not due to cessation of expression of the transfected cDNA, since expression of ICAM-2ΔCD protein persisted until time of death.

Because cell adhesion molecules and the actin cytoskeleton have been reported to regulate tumorigenic potential, we injected SK-N-ASpIRESneo and SK-N-ASpIRES.ICAM2 cells subcutaneously into SCID mice (5 mice per group, bilateral flank injections) and observed the mice for tumor development. Both cell types produced subcutaneous tumors within the same time frame (Fig. 4A). Subcutaneous local tumor growth was rapid and mice were euthanized for humane reasons before metastases were observed. Since both ICAM-2 and control transfectants readily formed subcutaneous tumors, we concluded that ICAM-2 was unlikely to be acting as a tumor suppressor. However, data from *in vitro* experiments showed that ICAM-2 expression was coincident with actin fiber redistribution and inhibition of cell motility. Since cell motility and the consequent migratory and invasive potential are essential to the metastatic process, we used a preclinical model of metastatic neuroblastoma to investigate whether ICAM-2 expression affected the development of disseminated tumors in SCID mice. In this model of metastatic neuroblastoma there is no primary tumor per se; but tumor cells injected intravenously must migrate through the endothelial cell monolayer of vasculature and invade stromal tissue to seed metastatic tumor foci and facilitate tumor progression.

Mice injected intravenously with SK-N-ASpIRESneo cells (or other neuroblastoma cell lines) develop tumors at multiple anatomic locations and require euthanasia within 2–3 months (Fig. 4B, left panels) due to progressive disease [33]. In contrast, mice receiving SK-N-ASpIRES.ICAM2 cells intravenously developed no detectable tumors for >12 months and survived significantly longer than mice receiving SK-N-ASpIRESneo cells (*P*<0.001) (Fig. 4C). These data showed that ICAM-2 abrogated the ability of SK-N-AS cells to develop disseminated neuroblastoma tumors in SCID mice. In contrast to SK-N-ASpIRES.ICAM2 cells, SK-N-ASpIRES.ICAM2ΔCD cells produced disseminated tumors (Fig. 3C) despite persistent expression of this truncated protein (Fig. 3D). We concluded that ICAM-2-mediated effects in this model depended on an intact intracellular domain. Together, *in vitro* and *in vivo* data suggest that the observed nonmetastatic phenotype could be due, at least in part, to the interaction of membrane-bound ICAM-2 with the cytoskeletal linker protein α-actinin.

**Figure 5:**
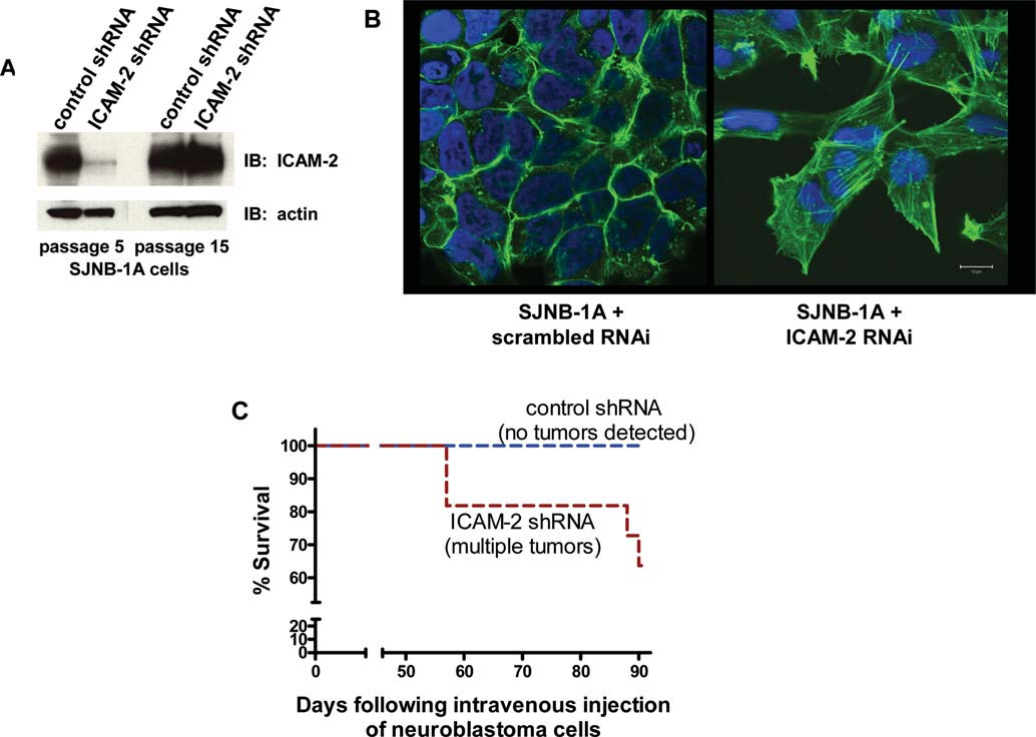
RNAi techniques indicated that the observed ICAM-2-induced phenotype was unlikely to be due to artificial overexpression of this protein. A) SJNB-1A cells express a relatively high endogenous level of ICAM-2 (Fig. 1A). The expression of ICAM-2 was inhibited in a population of SJNB-1A cells transfected to stably express ICAM-2 shRNA. This inhibition was transient even in the presence of selection pressure by G418 *in vitro*. B) Consistent with the proposed hypothesis, the high endogenous level of ICAM-2 expression in SJNB-1A cells is co-incident with a juxtamembrane distribution of actin fibers (left panel); and when ICAM-2 expression is decreased, actin fibers are transverse (right panel). C) Inhibition of ICAM-2 expression increased the development of disseminated neuroblastoma tumors in SCID mice injected intravenously with SJNB-1A ICAM-2 shRNA cells.

To address the possibility that the observed results were a consequence of artificial overexpression of ICAM-2, we used RNAi techniques to decrease the expression of ICAM-2 protein in SJNB-1A cells, the neuroblastoma cell line expressing the highest endogenous level of ICAM-2 expression (Fig. 1A) and investigated the phenotype of RNAi transfectants. At early passages, stably transfected SJNB-1A cells expressed <10% of the level of ICAM-2, compared to control transfectants (Fig. 5A). Consistent with previous results, control siRNA-transfected SJNB-1A cells had a juxtamembrane distribution of actin fibers (high ICAM-2::juxtamembrane actin fibers) (Fig. 5B) whereas siRNA transfected SJNB-1A cells had transverse actin fibers (low ICAM-2::transverse fibers). In addition, mice injected intravenously with control transfected SJNB-1A cells (high ICAM-2) developed no detectable tumors, while 40% of mice (N = 10/group) injected with ICAM-2 shRNA-transfected cells developed multiple tumors (Fig. 5C). While we would have expected 100% of the mice receiving ICAM-2 shRNA-transfected cells to develop tumors, we note that even in the presence of selection pressure *in vitro*, the suppression of ICAM-2 expression did not persist long-term (Fig. 5A, passage 15), and results in Fig. 5C are consistent with the transient downregulation of expression seen for ICAM-2 in SK-N-AS ICAM-2 shRNA cells. These results indicate that observations using neuroblastoma cells transfected to increase expression of ICAM-2 are unlikely to be due to artificial over-expression of this protein, since downregulation of endogenous ICAM-2 in SJNB-1A cells produced the expected phenotype: an inverse relationship between level of ICAM-2 expression with actin fiber redistribution and development of disseminated tumors *in vivo* was seen in both cDNA and RNAi transfected cell lines.

### In primary neuroblastoma tumors, as well as in cell lines, ICAM-2 expression and metastatic potential are inversely related

We hypothesized that if the above observations reflected the biology of primary tumors, ICAM-2 would be expressed by primary neuroblastoma cells with limited potential for development of progressive metastatic disease. This hypothesis was investigated by immunostaining for ICAM-2 of a neuroblastoma tissue microarray (TMA) obtained from the Children's Oncology Group. The TMA contained 90 independent patient samples, of which 89 samples gave interpretable results.

**Figure 6:**
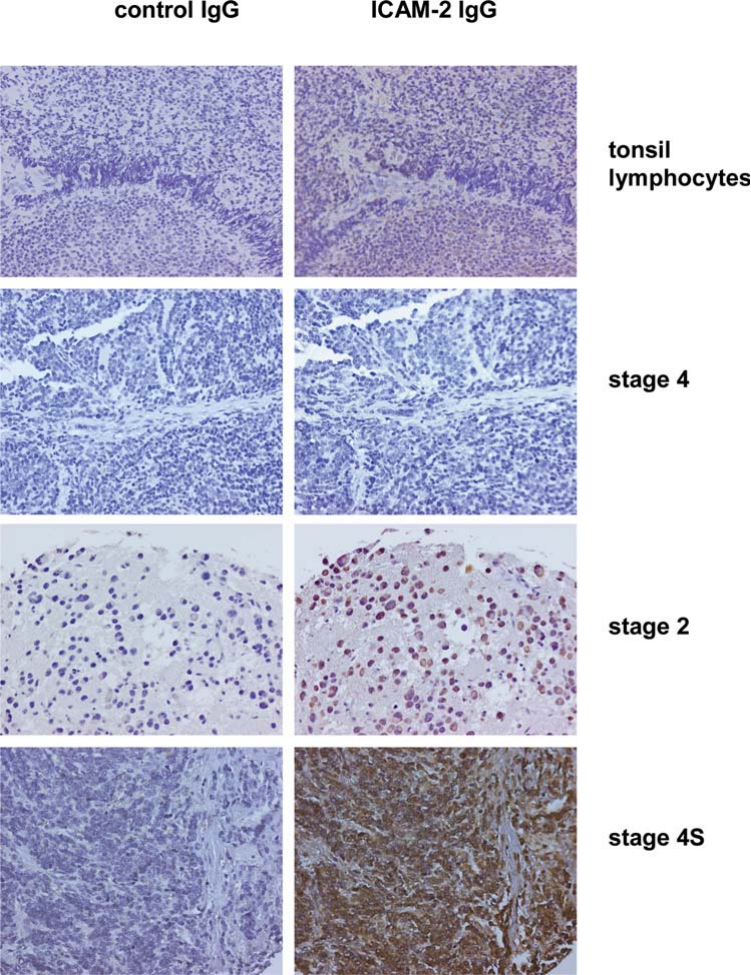
Immunohistochemical analyses ICAM-2 expression with a tissue microarray containing primary tumor specimens from 90 individual patients showed that a relatively high level of ICAM-2 expression was associated with primary tumor cells recognized to have limited metastatic potential. ICAM-2 antibody binding was detected using a peroxidase-tagged secondary antibody and DAB substrate (brown), by standard methods. Specimens were counterstained with hematoxylin/eosin, to visualize morphology of tumor and stromal cells. Staining was scored as: percent of stained cells×staining intensity (0, 1+, 2+, or 3+). The scores for ICAM-2 expression of the Stage 4, Stage 2, and Stage 4S tumor specimens pictured were 0, 270, and 300, respectively. Normal lymphocytes (a positive control) received a score of 100 (100% of lymphocytes×a staining intensity of 1+). Each specimen is described in more detail in the text.

Photomicrographs of representative tumor specimens demonstrating the range of staining intensity of primary tumor samples are shown in Fig. 6. The left panels of this Figure show immunoperoxidase staining with a control rabbit polyclonal antiserum and are appropriately negative. Of the right panels, the top panel shows a weak (1+) but unequivocal level of ICAM-2 expression by 100% of normal lymphocytes infiltrating human tonsilar tissue (Expression Score = 100%×1+ = 100). Lymphocytes are one of the few types of normal cells that express ICAM-2 protein, and the photomicrograph reflects the known low-level expression that has been reported for the few normal cells that do express ICAM-2 [19].

The three lower right-hand panels demonstrate ICAM-2 staining in representative stage 4, stage 2, and stage 4S neuroblastoma tumors. The stage 4 tumor is a high-risk, poorly differentiated, Schwannian stroma-poor tumor comprised of immature neoplastic cells. Patients with tumors of this stage and histology have a poor prognosis with virtually certain development of metastatic disease. Consistent with results obtained using neuroblastoma cells lines, ICAM-2 expression (Expression Score = 0) and metastatic potential are inversely related. The stage 2 tumor is a differentiating neuroblastoma, Schwannian stroma-poor, comprised of some immature neoplastic cells (∼10% of tumor cells) as well as a sizeable population of neoplastic cells that exhibit histologic features of ganglionic differentiation (∼90% of tumor cells). The immature neuroblastoma cells are negative for ICAM-2, but all tumor cells exhibiting histologic features of ganglionic differentiation express high levels (3+) of ICAM-2. The overall score for this tumor specimen is 90%×3+ = 270. The third example is a stage 4S tumor, which represents a special subtype of neuroblastoma with favorable prognosis [34]. It is defined as neuroblastoma arising in a child younger than 18 months with limited tumor spread to the skin and/or liver that is amenable to surgical resection. Despite its favorable prognosis, stage 4S neuroblastoma is comprised of immature neuroblastoma cells that are microscopically indistinguishable from immature cells seen in high-risk neuroblastoma tumors.

The immature neuroblasts (100%) of this stage 4S tumor exhibit strong (3+) ICAM-2 expression (Expression Score = 100%×3+ = 300), again reflecting a correlation between relatively high level of expression of ICAM-2 and low potential for progressive metastatic disease. Notably, the contrast between results for ICAM-2 expression of immature neuroblasts in the stage 4 tumor compared to the 4S tumor indicates that ICAM-2 is not simply a differentiation marker.

### Correlation between ICAM-2 expression and favorable clinicopathologic features

While long-term survival data are not yet available for the specific patients whose tumors are included in the COG microarray, the outcome for neuroblastoma patients can be reasonably predicted on the basis of well-characterized criteria that include histology, amplification status of the *MYCN* gene, age, and stage [28]. Patients with favorable histology low risk tumors have a greater than 95% overall survival with no adjuvant therapy beyond surgery. In contrast, patients with unfavorable histology and *MYCN*-amplified tumors have a ∼20% 5-year survival despite intensive therapy. Each tumor in the TMA was designated to have a “good” or “poor” prognosis based on histopathology, age at diagnosis, *MYCN* copy number status, and stage. The first three parameters were assessed in line with the criteria adopted by the International Neuroblastoma Pathology Committee, based on the Shimada classification [35]. With respect to histopathology, the most immature primary neuroblastoma cells (neuroblasts) are characterized by small size and scant cytoplasm. This undifferentiated cell type is usually associated with high-risk disease and less favorable clinical outcome. On the other extreme are cells with obvious advanced ganglionic differentiation, with abundant cytoplasm that includes a prominent basophilic golgi body area and a round eccentric nucleus with a prominent nucleolus. Primary tumor cells exhibit varying degrees of ganglionic differentiation. Cells with advanced ganglionic differentiation are associated with lower-risk disease and favorable clinical outcome. The presence of abundant stromal tissue (Schwannian stroma), is also usually associated with a more favorable prognosis. These criteria, in conjunction with age and tumor staging information provided by the Children's Oncology Group Biological Studies Oversight Committee formed the basis of designation of tumors in Table 1 as having “good” or “poor” prognosis.

**Table 1:** Characteristics of tumors evaluated for ICAM-2 expression.

N	ICAM-2 score <30	ICAM-2 score ≥30 & ≤90*	ICAM-2 score ≥160 & ≤300
	52	13	24
**Gender**			
Male	32 (62%)	9 (69%)	16 (67%)
Female	20 (38%)	4 (31%)	8 (33%)
**Race**			
White	45 (87%)	9 (69%)	19 (79%)
Black	1 (2%)	1 (8%)	2 (8%)
Hispanic	2 (4%)	0 (0%)	0 (0%)
Filipino	1 (2%)	0 (0%)	0 (0%)
Unknown or other	3 (6%)	3 (23%)	3 (13%)
**Age**			
<1 year	25 (48%)	4 (31%)	13 (54%)
≥1 year - ≤18 months	3 (6%)	0 (0%)	0 (0%)
>18 months	24 (46%)	9 (69%)	11 (46%)
**Stage**			
1	5 (10%)	3 (23%)	3 (13%)
2/2A/2B	9 (17%)	2 (15%)	3 (13%)
3	8 (15%)	3 (23%)	2 (8%)
4	18 (35%)	4 (31%)	9 (38%)
4S	9 (17%)	1 (8%)	6 (25%)
Stage not available	1 (2%)	0 (0%)	0 (0%)
N/A (GN patients)	2 (4%)	0 (0%)	1 (4%)
**Shimada Pathology**			
Favorable Histology/GN	17 (33%)	5 (38%)	7 (29%)
Unfavorable Histology	19 (37%)	7 (54%)	10 (42%)
Post-Treatment	3 (6%)	0 (0%)	3 (13%)
Post-Treatment (UH by age)	1 (2%)	0 (0%)	0 (0%)
Unknown/missing	12 (23%)	1 (8%)	4 (17%)
**Prognosiŝ**			
Good			**19 (83%)**
Poor			**4 (17%)**
**ICAM-2 expressed by:**			
Differentiated ganglionic cells		4 (31%)	15 (63%)
Both differentiated cells and undifferentiated neuroblasts	n/a	6 (46%)	6 (25%)
Neuroblasts		3 (23%)	3 (13%)

* No specimens received a score of >90 & <160. ˆ As defined in References 28, 34, and 35, and explained in the text.

A summary of the results for the staining of the 89 interpretable specimens (Table 1) showed that 42% (37 of 89) of primary tumors expressed ICAM-2 and that 65% of these (24 of 37) expressed ICAM-2 at relatively high levels (a score of >150 of a possible 300). Interestingly, all 89 samples scored either ≤90 or ≥160, with no intermediate results. Consistent with the preclinical data from neuroblastoma cell lines, ICAM-2 expression was associated with favorable histologic features (exact 95% confidence interval, 62.6% to 95.3%) such as ganglionic differentiation or low-risk stage [34], [35]. Preclinical and TMA data support the hypothesis that ICAM-2 is associated with a nonmetastatic phenotype in primary neuroblastoma cells or cell lines.

Consistent with our hypothesis, we observed the highest levels of ICAM-2 expression in neuroblastoma tumor cells associated with the most favorable predicted clinical outcome. Namely, tumor cells exhibiting histologic evidence of ganglionic differentiation were the predominant subset of ICAM-2-positive cells.

## Discussion

The novel findings of this study are that neuroblastoma cells express ICAM-2 and that this expression is associated with juxtamembrane distribution of actin fibers, decreased cell motility *in vitro*, and suppressed development of disseminated tumors in a preclinical model of metastatic neuroblastoma. These functions for ICAM-2 were independent of immune stimulation, since the tumorigenic potential of ICAM-2-expressing neuroblastoma cells as xenografts in SCID mice was unaffected and cell phenotype was altered *in vitro* as well as *in vivo*. Results with primary tumor specimens also reflected the inverse relationship between ICAM-2 expression and predicted low-risk for progressive metastatic disease, as was seen with the preclinical model.

Our data are in agreement with published literature in that the novel function reported here for ICAM-2 is similar to that of the related cell adhesion molecule CEACAM1 which binds directly to actin to inhibit tumorigenesis [36], but without the requirement for a cytoplasmic linker protein. ICAM-2 and CEACAM1 are also similar in that their association with the actin cytoskeleton and their suppression of tumor growth or metastasis (for CEAMCAM1 or ICAM-2, respectively) depend on an intact intracellular domain [37 and data herein].

Multiple other functions have been reported for ICAM-2 in different cell types. For example, gastric carcinoma tumor cells transfected to express ICAM-2 induce an immune response sufficient to produce a significant anti-tumor effect in immune competent mice [22]. Potentially, the dual functions of stimulating an immune response and inhibiting tumor cell motility could contribute to a more favorable clinical outcome. ICAM-2 has also been reported to increase the motility of endothelial cells and leukocytes, and also to activate AKT leading to inhibition of apoptosis [24]–[27]. Interestingly, ICAM-2 expression facilitated rather than inhibited endothelial cell motility. The increase in endothelial cell motility was directly related to an increased phosphorylation of Rac, a small guanosine triphosphatase (GTPase) and known regulator of actin organization, cell motility, and angiogensis [27]. ICAM-2 also facilitated leukocyte migration, albeit in a stimulus-specific manner [24]. Also in contrast to published studies with osteosarcoma and breast cancer cells [10], [11], we detected no involvement of the linker protein ezrin in the phenotype that ICAM-2 conferred on neuroblastoma cells.

The as yet unexplained differences in function of ICAM-2-interacting proteins in different cell types are, nonetheless, relatively common observations. Disease progression in uveal malignant melanoma [38] is associated with a relatively high level of expression of the ERM cytoskeletal linker protein ezrin. Conversely, disease progression is associated with a relatively low level of expression of this protein in serous ovarian carcinoma [39]. Similarly, the linker protein α-actinin-4 decreases the tumorigenic potential of neuroblastoma cells [7] but is associated with a progressive phenotype in breast and colorectal cancer [4], [40].

We speculate that varying levels of expression of ICAM-2-interacting proteins by specific cell types or the protein composition of extracellular matrices or of adjacent cells in different tissues may modulate the state of activation (conformation) and/or the function of ICAM-2. In tumor cells in particular, constitutive activation of proteins up- or down-stream of the bidirectional signals mediated by CAMs might also affect ICAM-2 function and cell phenotype. Quite likely, while some of the protein interactions of ICAM-2 and the pathways or functions affected by these interactions are known (e.g., binding of ICAM-2 to CD11a/CD18 and CD11b/CD18 integrins facilitates adhesion of human leukocytes to endothelial cells [20]; and ICAM-2 induces phosphorylation of Rac and modulates motility of endothelial cells [27]), others will undoubtedly be identified.

However, despite the multiple proteins and pathways known to be affected by cell adhesion molecules in general and ICAM-2 in particular, complete membrane-to actin linkages that regulate CAM function in tumor cells have been difficult to identify. Several laboratories, however, have described the involvement of specific linker proteins and documented the impact of these linker proteins on the migratory and metastatic potential of tumor cells. In leukemic cells, for example, membrane-bound tetraspanin proteins EWI-2 and EWI-F bind to cytoskeletal linker ezrin-radixin-moesin (ERM) proteins to regulate cell migration [12]. This binding links cell membranes (EWI-1 and EWI-F proteins) with cytoplasmic actin cytoskeletal proteins (ERM proteins), but simultaneous association of these complexes with actin fibers has not yet been reported. Also of note, individual linker proteins appear to function differently in cells from different tumor types.

In neuroblastoma cells, we detected an association of ICAM-2 with α-actinin, and data with competitive peptides and truncated proteins suggest that this association plays a role in the regulation of the observed ICAM-2-mediated phenotype. Potentially, in addition to limiting the interaction of ICAM-2 with α-actinin, truncation of the intracellular domain of ICAM-2 may also have prevented the association of membrane-bound ICAM-2 with other as-yet-unidentified intracellular proteins. However, the hypothesis that the novel function for ICAM-2 described here depends at least partly on the association of membrane-bound ICAM-2 with α-actinin and actin is consistent with published reports documenting the role of α-actinin as a determinant of metastatic potential [4], [5], [40], [41] and the observation that α-actinin associates with an unknown membrane protein to decrease the tumorigenic or metastatic potential of neuroblastoma cells [7]. Our data indicate that ICAM-2 may be such a membrane protein. Our results are also consistent with the correlations between the juxtamembrane localization of actin fibers and decreased motility or decreased metastatic potential that have been reported for mouse embryonic fibroblasts [42] and for primary oral squamous cell carcinoma tumors [43], respectively.

Finally, the finding that ICAM-2 expression inhibited tumor cell motility in scratch and modified Boyden chamber invasion assays *in vitro* merits further discussion. With respect to metastasis, *in vitro* cell motility assays may indicate how likely a tumor cell is 1) to detach from a primary tumor (and enter systemic circulation); or 2) to migrate through the vascular endothelial layer at an anatomic site distant from the primary tumor; or, 3) once through the endothelium, to invade the normal tissue stroma to facilitate development of tumor at secondary sites.

We suggest that the component of the metastatic process most likely affected by ICAM-2 is the invasion of normal stroma. This opinion is based primarily on results from immunostaining of the TMA, and from scratch and Boyden chamber assays. Firstly, ICAM-2 was expressed predominantly by primary neuroblastoma cells with some degree of ganglionic differentiation, and therefore predicted low metastatic potential. The notable exception to this was the 3+ staining of undifferentiated neuroblasts of some 4S tumors. Patients with 4S tumors frequently have liver, dermal, and/or limited (<10%) bone marrow, as well as adrenal, involvement at diagnosis, i.e., secondary tumors are present at diagnosis. However, unlike stage 4 tumors, 80% of patients with 4S disease do not develop progressive disease at metastatic sites. Rather, 4S tumors usually regress spontaneously without treatment, suggesting a limited invasive potential. Secondly, scratch assays performed in the presence of Matrigel, in which extracellular matrix proteins can influence cell function, showed readily distinguishable differences between control and ICAM-2 transfectants. However, when these assays were performed in the absence of Matrigel (data not shown), no differences in cell motility were observed. Thirdly, results from standard Boyden chamber invasion assays, in which cells invade a layer of Matrigel to migrate toward a chemoattractant, corroborated results of scratch assays. The presence of extracellular matrix proteins *in vitro* influenced ICAM-2-mediated cell function. Fourthly, Stage 4 neuroblastomas are progressively invasive tumors with a generally poor outcome; while most Stage 4S tumors are self-limiting variants of neuroblastoma despite transient involvement of skin, liver, or bone marrow. The data are consistent with the hypothesis that ICAM-2 limits the invasive potential of neuroblastoma cells.

We suggest further that the significance of ICAM-2 expression can be compared with that of *MYCN* amplification. If *MYCN* is amplified, it is regarded as a prognostic indicator for poor outcome. If *MYCN* is not amplified, prognosis may still be poor—normal *MYCN* copy number is uninformative [28]. Similarly, ICAM-2 expression is associated with a more favorable prognosis. However, lack of ICAM-2 expression is not necessarily associated with poor prognosis; it is simply uninformative.

In summary, the observed ICAM-2/α-actinin/actin interaction appears to represent a complete membrane-to-actin linkage that contributes to regulation of the motility (possibly, the invasive potential) and therefore the metastatic phenotype of neuroblastoma cells. Our long-range goal is to determine the specific protein domains essential to this regulation and to investigate the possibility that small molecule agonists can be developed to mimic the effects of ICAM-2 expression in neuroblastoma cells.

## Materials and Methods

### Immunoprecipitations and Immunoblots

Immunoprecipitations of ICAM-2, α-actinin, ezrin, and actin done with antibodies IC2/2, MAB1682 (Chemicon International, Temecula, CA), C-15 (Santa Cruz Biotechnologies), and 4968 (Cell Signaling Technology), respectively. Immunoblots for ICAM-2, α-actinin, ezrin, actin, AKT, and phospho-AKT were done with AF244 (R&D Systems, Minneapolis, MN) and C-20 (Santa Cruz Biotechnologies, Beverly, MA), CBL231 (Research Diagnostics, Inc., Flanders, NJ), C-20 and C-15 (Santa Cruz Biotechnologies), 4968, 9272, and 4051 (Cell Signaling Technology), respectively. AKT and phospho-AKT standards were from Cell Signaling Technology. Isotype-matched control antibodies were from Jackson Immunolabs, Inc. (West Grove, PA).

### Cell Motility Assays

***Modified Boyden chamber invasion assays*** were done by standard methods [44]. Briefly, cells were overlaid on “solidified” Matrigel (upper chamber), with a chemoattractant (lower chamber) comprised of 10% fetal bovine serum in Dulbecco's Minimum Essential Medium supplemented with fibronectin (20 µg/mL) Cells that migrated through (invaded) the Matrigel layer and to the distal side of a membrane containing 8 micron pores were recorded as photomicrographs using standard inverted microscopy, and quantitated using an AlphaEase FC software (Alpha Innotech, San Leandro, CA). Results were expressed as the mean percentage of dark pixels/total pixels in 10× fields+S.D. from a minimum of five fields per experiment, of six replicate experiments.

***“Scratch” or “wound healing” assays*** were also done by standard methods. In brief, cell were plated in chamber well slides and cultured in Dulbecco's Minimum Essential Medium supplemented with 10% fetal bovine serum until confluent. A 200 microliter pipet tip was used to “wound” the confluent monolayers, which were immediately overlaid with 25% Matrigel. As soon as the Matrigel solidifed, photomicrographs were taken using an inverted microcroscope. Images were recorded at 0, 15, 24, and 48 hours after scratch.

### Plasmids Containing ICAM-2 cDNA

The cDNA of ICAM-2 was isolated from human umbilical vein cell RNA (Clonetics, San Diego, CA). Amplification was performed at 95°C for 1 min, then 30 cycles of 94°C for 30 sec, 55°C for 30 sec, 72°C for 1 min, and 72°C for 7 min. Primers for full length ICAM-2 were: forward (5′GCTTCCGCTGCCTGGATTCT3′) and reverse (5′AAGTCCAGGTGTTGTATTC3′), and for truncated ICAM-2: forward (5′ACGCAGGATCCGCCAGAGATGTCCTCTTTCG) and reverse (TTGGATCCTGATGAAGCAGAGCAGGACAGATG). cDNAs inserted into pIRESneo2 (Clontech, San Jose, CA) were transfected with FuGene6 (Roche Diagnostics, Indianapolis, IN), and transfectants selected with G418. The St. Jude Hartwell Center for Bioinformatics sequenced all plasmids.

### RNAi-mediated decrease in endogenous ICAM-2 expression

The siRNA or shRNA sequence used to decrease expression of endogenous ICAM-2 (CAAGAAGCTGGCGGTTGA) was designed using Invivogen siRNA Wizard and Dharmacon siDesign Center. siRNA sequences for transient transfections were synthesized in the St. Jude Hartwell Center for Technology. shRNA for stable transfections were done using psiRNA-hH1-ICAM2 (Invivogen). In this plasmid, shRNA expression is regulated by the H1 promoter, and neomycin resistance gene is regulated by expression of the SV40 promoter. Transfections were done with FuGene6 (Roche Applied Science, Indianapolis, IN). Stably transfected cells were selected using G418, and the entire population of G418-resistant cells was used for *in vivo* experiments. Control transfections were done with transfection reagent only or with scrambled RNAi and transfection reagent. Specificity of RNAi was assessed by probing immunoblots of RNAi and control transfectants for a minimum of three additional, irrelevant proteins.

### Competitive peptides that inhibit ICAM-2/α-actinin or α-actinin/actin binding

Peptides comprised of amino acids that mediate binding of ICAM-2 to α-actinin or of α-actinin to actin, or scrambled control peptides were linked to the antennapedia 16-mer that facilitates intracellular peptide accumulation. The antennapedia 16-mer (RQIKIWFQNRRMKWKK [45] was coupled to the 5′-end of each of the following sequences: VRAAWRRL (ICAM-2 amino acids 241-248 to inhibit ICAM-2/α-actinin binding [30]); LARRRWAV (scrambled control); MTLGMIWTIILRFA (α-actinin amino acids 122–135 to inhibit α-actinin/actin binding [46]); WIRMIAFGLIMTLT (scrambled control). Oligopeptides were synthesized in the St. Jude Hartwell Center for Bioinformatics and were >99% pure. SK-N-ASpIRES.ICAM2 cells were incubated with 12.5 µM peptide for 4 hours, at which time cells were fixed with paraformaldehyde and the subcellular localization of actin fibers evaluated using FITC-conjugated phalloidan by standard methods.

### Distribution of F-actin

Standard methods were used to fix cells using paraformaldehyde and incubate them for 30 minutes at room temperature with 10 µg fluorescein isothiocyanate (FITC)-conjugated phalloidan/mL phosphate buffered saline. Actin fibers were visualized by confocal fluorescence microscopy, also by standard methods.

### Mouse Models of Neuroblastoma

Subcutaneous xenografts: 1×10^7^ neuroblastoma cells transduced to express luciferin were injected subcutaneously in each hind flank of SCID mice (5 mice/group), and imaged noninvasively by standard methods. Disseminated neuroblastoma: 5×10^5^ neuroblastoma cells injected intravenously into the tail veins of SCID mice produced disseminated tumors in 100% of mice [33]. In this model, there is no “primary tumor”, but tumor development mimics that of clinical metastatic neuroblastoma in that tumors develop in multiple anatomic locations including supra-renal and bone marrow. Animal protocols were in accordance with the guidelines of the St. Jude Animal Care and Use Committee.

### Immunostaining primary tumor samples for ICAM-2

The neuroblastoma tissue microarray obtained from the Children's Oncology Group Neuroblastoma Biology Committee, Children's Hospital of Philadelphia included triplicate cores from 90 distinct patients at presentation: 47 low-risk group (16 Stage 1, 16 Stage 2, 15 Stage 4S); 43 high-risk group (15 Stage 3, 28 Stage 4) [28].

Sections were incubated overnight with polyclonal antiserum to ICAM-2 (ProteinTech Group, Inc., Chicago, IL), and antibody binding detected with biotinylated secondary antibody and ABC Reagent (Vectastain ABC Kit, Vector Laboratories, Burlingame, CA). The primary antibody was not detected to cross react with any other protein, including ICAM-1. Results were scored blinded by JDK, according to the following criteria: staining intensity: 1, weak; 2, moderate; or 3, strong. The percentage of tumor cells positive for ICAM-2 was estimated by counting at least 500 tumor cells in each core. Scores = % positive cells×estimated intensity. The core with the highest score determined the final value for each tumor. A score ≤20 was considered negative.

### Statistical Analyses

With the exception of the tissue microarray, data were analyzed with GraphPad Prism software (San Diego, CA). Results for cell motility assays were expressed as the number of pixels occupied by Diff-Quick (IMEB Inc., San Marcos, CA)-stained cells on the distal side of membranes with 8 µm pores. Data were analyzed using a two-sided *t*-test. Migration assays were done at least three times, with six replicates of each cell line. Kaplan-Meier plots compared survival using a log-rank (Mantel-Haenszel) test.

## Supporting Information

Figure S1 (1.11 MB TIF)

Figure S2 (1.27 MB TIF)

## References

[1] Bretscher A (1999). Regulation of cortical structure by the ezrin-radixin-moesin protein family.. Curr Opin Cell Biol.

[2] Sun C-X, Robb VA, Gutmann DH (2002). Protein 4.1 tumor suppressors: getting a FERM grip on growth regulation.. J Cell Sci.

[3] Yamazaki D, Kurisu S, Takenawa T (2005). Regulation of cancer cell motility through actin reorganization.. Cancer Sci.

[4] Honda K, Yamada T, Endo R, Ino Y, Gotoh M (1998). Actinin-4, a novel actin-bundling protein associated with cell mobility and cancer invasion.. J Cell Biol.

[5] Gluck U, Ben-Ze'ev A (1994). Modulation of alpha-actinin levels affects cell motility and confers tumorigenicity on 3T3 cells.. J Cell Sci.

[6] Hunter KW (2004). Ezrin, a key component in tumor metastasis.. Trends Mol Med.

[7] Nikolopoulos SN, Spengler BA, Kisselbach K, Evans AE (2000). The human non-muscle α-actinin protein encoded by the ACTN4 gene suppresses tumorigenicity of human neuroblastoma cells.. Oncogene.

[8] Comoglio PM, Boccaccio C, Trusolino L (2003). Interactions between growth factor receptors and adhesion molecules: breaking the rules.. Curr Opin Cell Biol.

[9] Sampath R, Gallagher PJ, Pavalko FM (1998). Cytoskeletal interactions with leukocyte integrin beta2 cytoplasmic tail. Activation-dependent regulation of associations with talin and alpha-actinin.. J Biol Chem.

[10] Khanna C, Wan X, Bose S, Cassaday R, Olomu O (2004). The membrane-cytoskeleton linker ezrin is necessary for osteosarcoma metastasis.. Nat Med.

[11] Elliott BE, Meens JA, SenGupta SK, Louvard D, Arpin M (2005). The membrane cytoskeletal crosslinker ezrin is required for metastasis of breast carcinoma cells.. Breast Cancer Res.

[12] Sala-Valdes M, Ursa A, Charrin S, Rubinstein E, Hemler ME (2006). EWI-2 and EWI-F link the tetraspanin web to the actin cytoskeleton through their direct association with ezrin-radixin-moesin proteins.. J Biol Chem.

[13] Iino H, Fukayama M, Maeda Y, Koike M, Mori T (1994). Molecular genetics for clinical management of colorectal carcinoma: 17p, 18q, and 22q loss of heterozygosity and decreased DCC expression are correlated with metastatic potential.. Cancer.

[14] Thies A, Moll I, Berger J, Wagener C, Brummer J (2002). CEACAM1 expression in cutaneous malignant melanoma predicts the development of metastatic disease.. J Clin Oncol.

[15] Klaile E, Muller MM, Kannickt C, Singer BB, Lucka L (2005). CEACAM1 functionally interacts with filamin A and exerts a dual role in the regulation of cell migration.. J Cell Sci.

[16] Leung N, Turbide C, Olson M, Marcus V, Jothy S (2006). Deletion of carcinoembryonic antigen-related cell adhesion molecule 1 (Ceacam1) gene contributes to colon tumor progression in a murine model of carcinogenesis.. Oncogene.

[17] Reyes-Mugica M, Lin P, Yokota J, Reale M (1998). Status of deleted in colorectal cancer gene expression correlates with neuroblastoma metastasis.. Lab Invest.

[18] Carpenito C, Pyszniak AM, Takei F (1997). ICAM-2 provides a costimulatory signal for T cell stimulation by allogeneic Class II MHC.. Scand J Immunol.

[19] deFougerolles AR, Stacker SA, Schwarting R, Springer TA (1991). Characterization of ICAM-2 and evidence for a third counter-receptor for LFA-1.. J Exp Med.

[20] Kotovuori A, Pessa-Morikawa T, Kotovuori P, Nortamo P, Gahmberg CG (1999). ICAM-2 and a peptide from its binding domain are efficient activators of leukocyte adhesion and integrin affinity.. J Immunol.

[21] Reiss Y, Engelhardt B (1999). T cell interaction with ICAM-1-deficient endothelium in vitro: transendothelial migration of different T cell populations is mediated by endothelial ICAM-1 and ICAM-2.. Int Immunol.

[22] Tanaka H, Yashiro M, Sunami T, Sakate Y, Kosaka K (2004). ICAM-2 gene therapy for peritoneal dissemination of a scirrhous gastric carcinoma.. Clin Cancer Res.

[23] Melero I, Gabari I, Tirapu I, Arina A, Mazzolini G (2003). Anti-ICAM-2 monoclonal antibody synergizes with intratumor gene transfer of interleukim-12 inhibiting activation-induced T-cell death.. Clin Cancer Res.

[24] Huang M-T, Larbi KY, Scheiermann C, Woodfin A, Gerwin N (2006). ICAM-2 mediates neutrophil transmigration in vivo: evidence for stimulus specificity and a role in PECAM-2-independent transmigration.. Blood.

[25] Perez OD, Kinoshita S, Hitoshi Y, Payan DG, Kitamura T (2002). Activation of the PKB/AKT pathway by ICAM-2.. Immunity.

[26] Singh K, Colmegna I, He X, Weyand CM, Goronzy JJ (2008). Synoviocyte stimulation by the LFA-1-intercellular adhesion molecule-2-ezrin-Akt pathway in rheumatoid arthritis.. J Immunol.

[27] Huang M-T, Mason JC, Birdsey GM, Amsellem V, Gerwin N (2005). Endothelial intercellular adhesion molecule (ICAM)-2 regulates angiogenesis.. Blood.

[28] Maris JM, Shusterman S, Holland JF, Frei E, Kufe DW, Bast RC, Hait WN (2006). Neuroblastoma.. Cancer Medicine 7.

[29] Yonemura S, Hirao M, Doi Y, Takahashi N, Kondo T (1998). Ezrin/radixin/moesin (ERM) proteins bind to a positively charged amino acid cluster in the juxta-membrane cytoplasmic domain of CD44, CD43, and ICAM-2.. J Cell Biol.

[30] Heiska L, Kantor C, Parr T, Critchley DR, Vilja P (1999). Binding of the cytoplasmic domain of intercellular adhesion molecule-2 (ICAM-2) to α-actinin.. J Biol Chem.

[31] Nortamo P, Salcedo R, Timonen T, Patarroyo M, Gahmberg CG (1991). A Monoclonal antibody to the human leukocyte adhesion molecule intercellular adhesion molecule-2.. J Immunol.

[32] Kuhlman PA, Ellis J, Critchley DR, Bagshaw CR (1994). The kinetics of the interaction between the actin-binding domain of alpha-actinin and F-actin.. FEBS Lett.

[33] Thompson J, Guichard SM, Cheshire PJ, Richmond LB, Poquette CA (2001). Development, characterization, and therapy of a disseminated model of childhood neuroblastoma in SCID mice.. Cancer Chemother Pharmacol.

[34] Simon T, Spitz R, Faldum A, Hero B, Berthold F (2004). New definition of low-risk neuroblastoma using stage, age, and 1p and MYCN status.. J Pediatr Hematol Oncol.

[35] Schwab M, Shimada H, Joshi V, Brodeur GM, Kleihues P, Cavenee WK (2000). Neuroblastic tumours of adrenal gland and sympathetic nervous system.. Pathology and genetics of tumours of the nervous system.

[36] Chen CJ, Kirshner J, Sherman MA, Hu W, Hguyen T (2007). Mutation analysis of the short cytoplasmic domain of the cell-cell adhesion molecule CEACAM1 identifies residues that orchestrate actin binding and lumen formation.. J Biol Chem.

[37] Izzi L, Turbide C, Houde C, Kunath T, Beauchemin N (1999). cis-Determinants in the cytoplasmic domain of CEACAM1 responsible for its tumor inhibitory function.. Oncogene.

[38] Makitie T, Carpen O, Vaheri A, Kivela T (2001). Ezrin as a prognostic indicator and its relationship to tumor characteristics in uveal malignant melanoma.. Investigative Ophthalmol & Visual Scient.

[39] Moilanen J, Lassus H, Leminen A, Vaheri A, Butzow R (2003). Ezrin immunoreactivity in relation to survival in serous ovarian carcinoma patients.. Gynecologic Oncol.

[40] Honda K, Yamada T, Hayashida Y, Idogawa M, Sato S (2005). Actinin-4 increases cell motility and promotes lymph node metastasis of colorectal cancer.. Gastroenterology.

[41] Menez J, Chansac BLM, Dorothee G, Vergnon I, Jalil A (2004). Mutant alpha-actinin-4 promotes tumorigenicity and regulates cell motility of a human lung carcinoma.. Oncogene.

[42] James MF, Beauchamp RL, Manchanda N, Kazlauskas A, Ramesh V (2004). A NHERF binding site links the βPDGFR to the cytoskeleton and regulates cell spreading and migration.. J Cell Sci.

[43] Kobayashi H, Sagara J, Kurita H, Morifuji M, Ohishi M (2004). Clinical significance of cellular distribution of moesin in patients with oral squamous cell carcinoma.. Clin Cancer Res.

[44] Kleinman HK, Jacob K, Ausubel FM, Brent R, Kingston RE, Moore DD, Seidman JG (1998). Invasion assays.. Current Protocols in Cell Biology.

[45] Pietersz GA, Li W, Apostolopoulos V (2001). A 16-mer peptide (RQIKIWFQNRRMKWKK) from antennapedia preferentially targets the Class I pathway.. Vaccine.

[46] Kuhlman PA, Hemmings L, Critchley DR (1992). The identification and characterization of an actin-binding site in α-actinin by mutagenesis.. FEBS.

