# Supplementary figures and images for: ICAM-2 Expression Mediates a Membrane-Actin Link, Confers a Nonmetastatic Phenotype and Reflects Favorable Tumor Stage or Histology in Neuroblastoma
Source: PLoS One. 2008 Nov 3;3(11):e3629. doi: 10.1371/journal.pone.0003629 (PMC2575377; doi:10.1371/journal.pone.0003629)

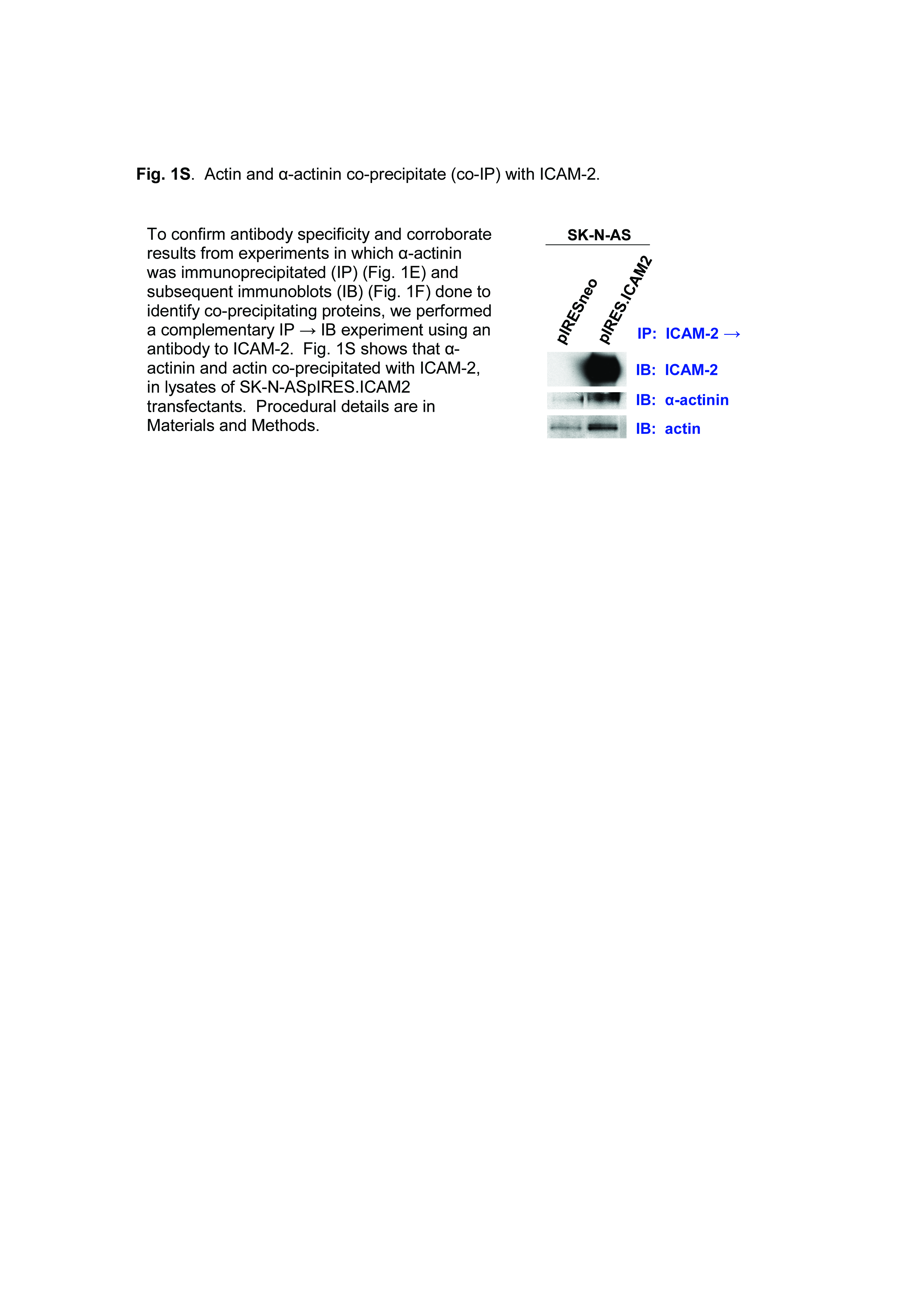

Supplement: Figure S1 — (1.11 MB TIF) [file pone.0003629.s001.tif]

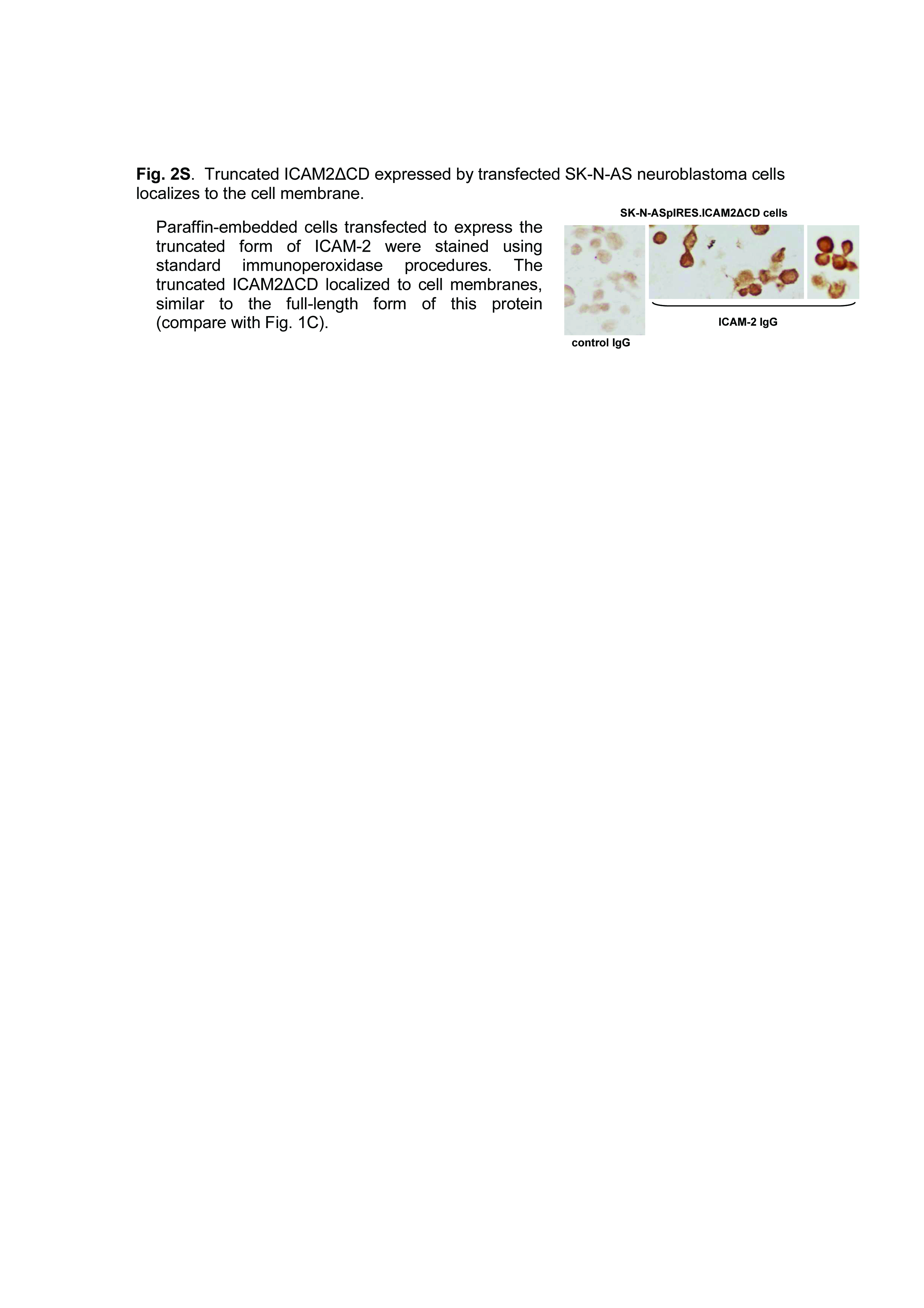

Supplement: Figure S2 — (1.27 MB TIF) [file pone.0003629.s002.tif]
